# Supplementary material for: Cell-autonomous immune gene expression is repressed in pulmonary neuroendocrine cells and small cell lung cancer
Source: Commun Biol. 2021 Mar 9;4:314. doi: 10.1038/s42003-021-01842-7 (PMC7943563; doi:10.1038/s42003-021-01842-7)
Supplement: Supplementary file 2 — Supplementary Information [file 42003_2021_1842_MOESM2_ESM.pdf]

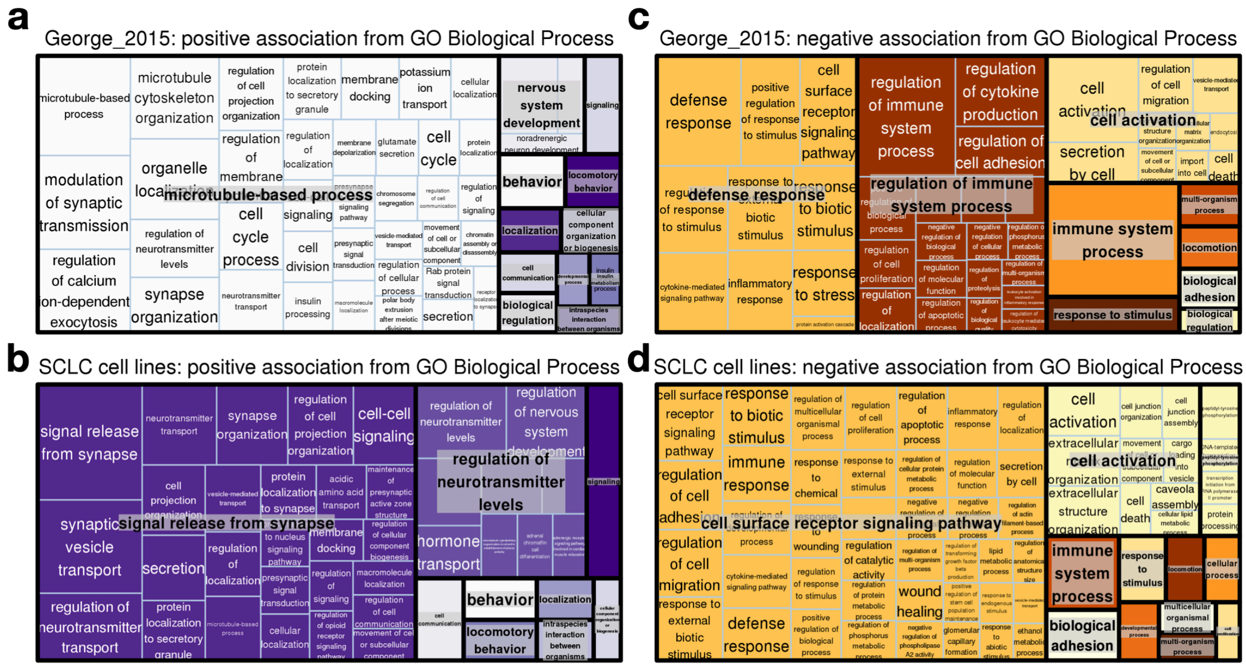

Supplementary Figure 1. Gene ontology analyses of genes associated with NE scores in SCLC datasets

REVIGO treemap summaries of enriched Biological Process GO terms for genes associated with NE scores in SCLC tumor dataset “George_2015” (**a** and **c**) or in SCLC cell lines (**b** and **d**). REVIGO algorithm was used to reduce redundancy in GO terms. Treemaps were used to visualize hierarchical structure among terms. Each rectangle with unique color/shade is a single cluster representative. The representatives are joined into “superclusters” of loosely related terms, visualized with rectangles with thicker borders. Size of the rectangles reflects p-values of associated terms.

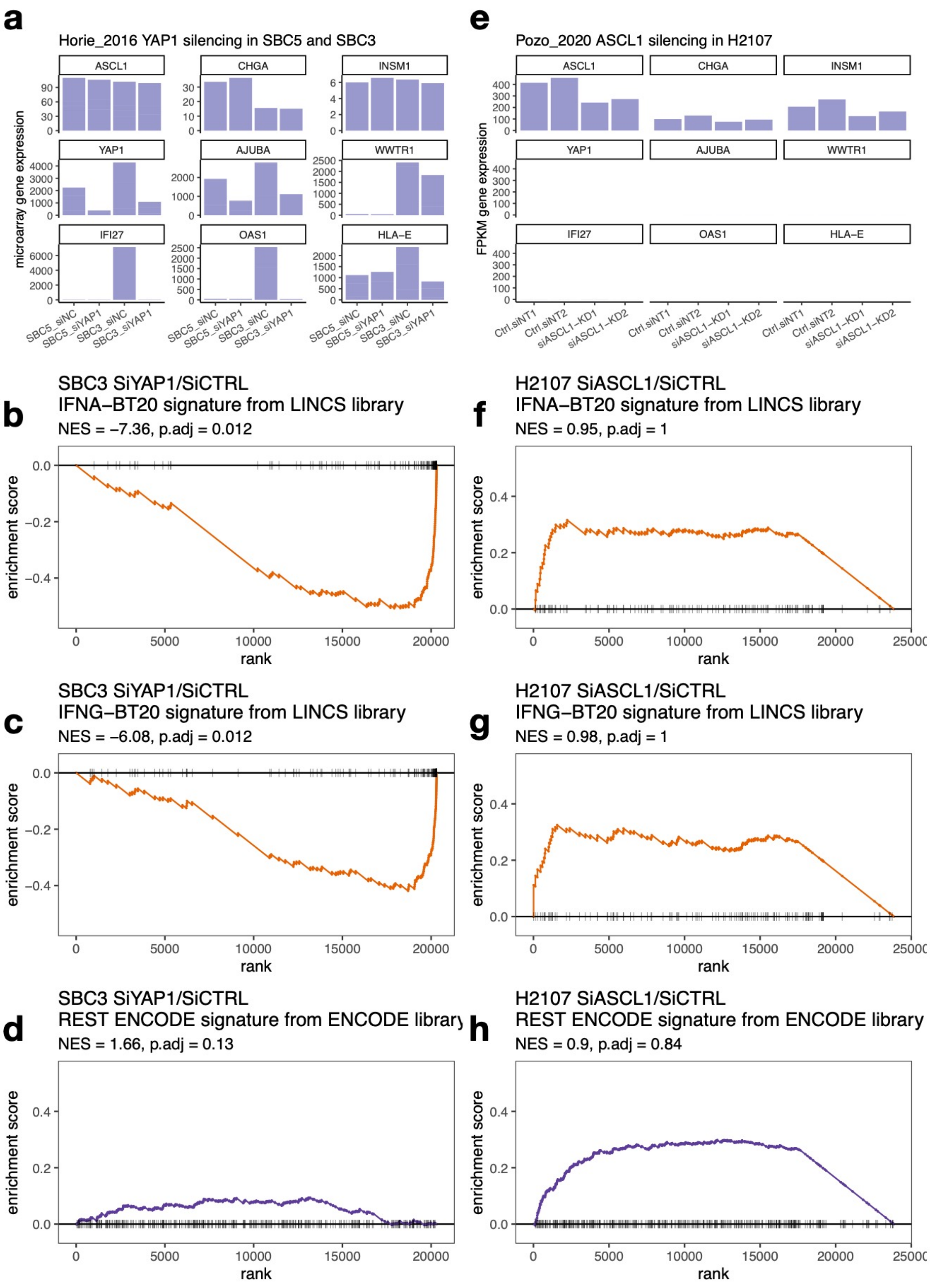

Supplementary Figure 2. YAP1 but not ASCL1 silencing alters expression of ISGs.
a, Expression of NE markers, Hippo pathway genes and ISGs in SBC3 and SBC5 with or without *YAP1* KD. Quantile normalized microarray data from GSE93400 deposited by Horie et al. was used to generate these bar plots. *ASCL1, CHGA* and *INSM1* are NE genes; *YAP1,*

AJUBA and *WWTR1* are Hippo pathway genes, *IFI27, OAS1,* and *HLA-E* are ISGs. YAP1 silencing did not alter NE markers genes. ISGs are repressed by YAP1 silencing in SBC3 but not SBC5 cell line. Note however, baseline levels of ISGs are lower in SBC5 and Hippo pathway gene *WWTR1* levels are also much lower in SBC5. **b**-**d**, Enrichment plot with GSEA statistics to examine how YAP1 silencing affect the expression of IFNA targets (**b**), IFNG targets (**c**) and REST targets (**d**) in SBC3 cells. P-values were estimated by permutations of gene labels. SiYAP1/SiCTRL fold change was used as the ranking metric (highest to lowest from left to right) for GSEA. Normalized enrichment plot (NES) and multiple comparison adjusted p-value is provided under the title of each subplot. **e**, Expression of NE markers, Hippo pathway genes and ISGs in H2107 with or without *ASCL1* KD. Quantile normalized RNA-seq FPKM data was used to generate the bar plots. **f**-**h**, Enrichment plot with GSEA statistics to examine how ASCL1 silencing affect the expression of IFNA targets (**f**), IFNG targets (**g**) and REST targets (**h**).

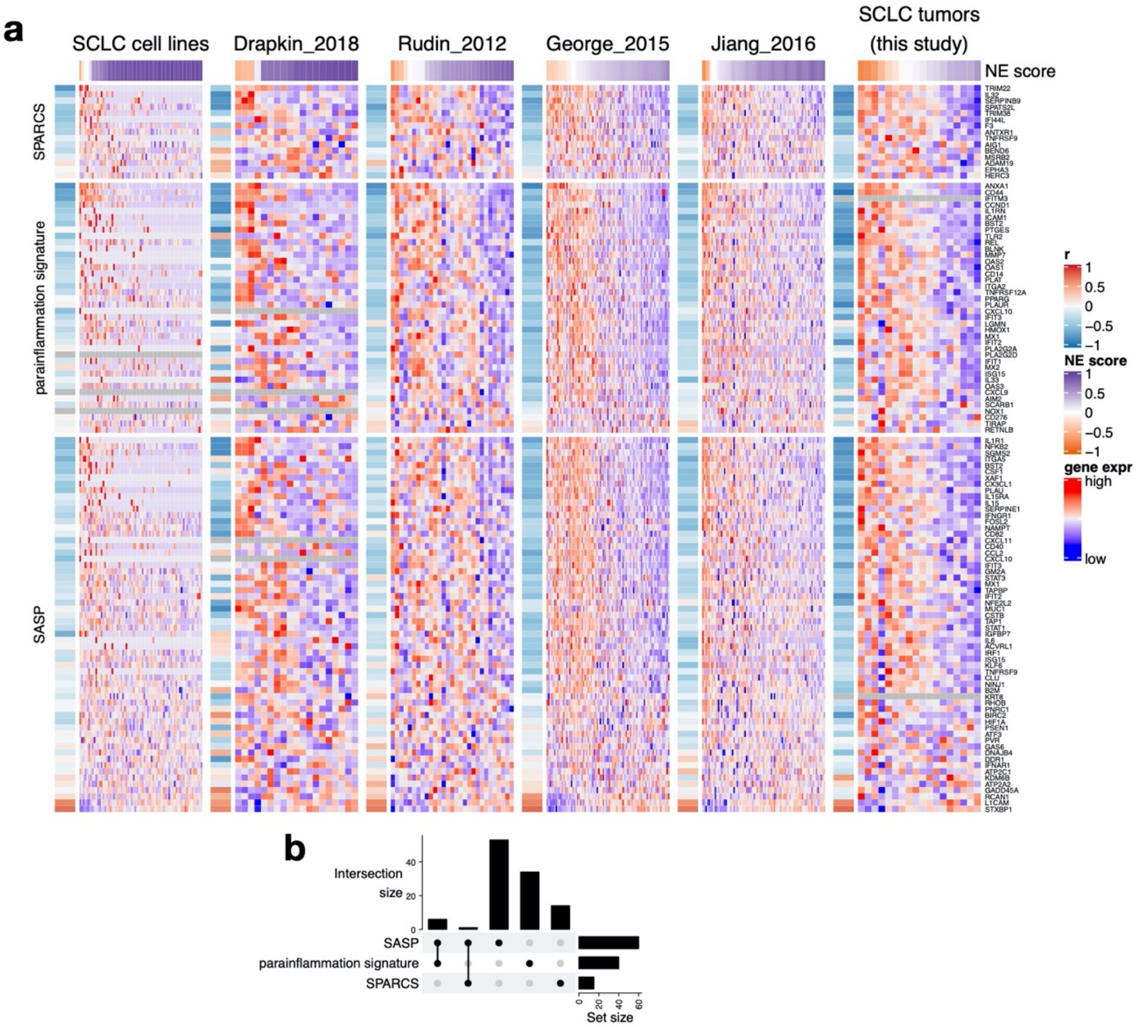

c SCLC cell lines

NE association	innate immune gene FALSE	TRUE	Row Total
negative	11837	508	12345
	45.747%	1.963%	
positive	13229	301	13530
	51.127%	1.163%	
Column Total	25066	809	25875

Pearson's Chi-squared test

Chi^2 = 76.15804 d.f. = 1 p = 2.6e-18

d NBL cell lines

NE association	innate immune gene FALSE	TRUE	Row Total
negative	11490	533	12023
	42.781%	1.985%	
positive	14464	371	14835
	53.854%	1.381%	
Column Total	25954	904	26858

Pearson's Chi-squared test

Chi^2 = 76.23645 d.f. = 1 p = 2.5e-18

Supplementary Figure 3. Expression of SPARCS, parainflammation and SASP genes in SCLC datasets

a, Heatmaps visualizing expression of selected immune gene sets in multiple SCLC datasets. These gene sets were previously reported in different studies to express cell autonomously in cancer. Genes within each gene set were ordered by correlation with NE score from meta-analysis of all datasets. Gene expression matrix of each dataset was annotated with a left-side column with color-coded Pearson correlation coefficient (from correlating NE score with gene expression), and a top bar indicating NE scores. Sample-wise z-score standardized expression

values were used for each dataset. **b**, UpSet plot showing gene counts in intersections of the three gene sets used in (**a**). SPARCS genes are genes with stimulated 3 prime antisense retroviral coding sequences; these genes have been shown to activate IFN-mediated innate immune pathways (1). Parainflammation genes are innate immunity genes that were found to express in cancer (2). SASP genes are senescence-associated secretory phenotype genes (3). **c-d**, Statistical tests showing negative correlations with NE scores are significantly more frequent for innate immune genes defined by innateDB(4), both in SCLC cell lines (**b**) and NBL cell lines (**c**). Full table and annotation of genes from innateDB and their correlations with NE scores is provided as Supplementary Table 4.

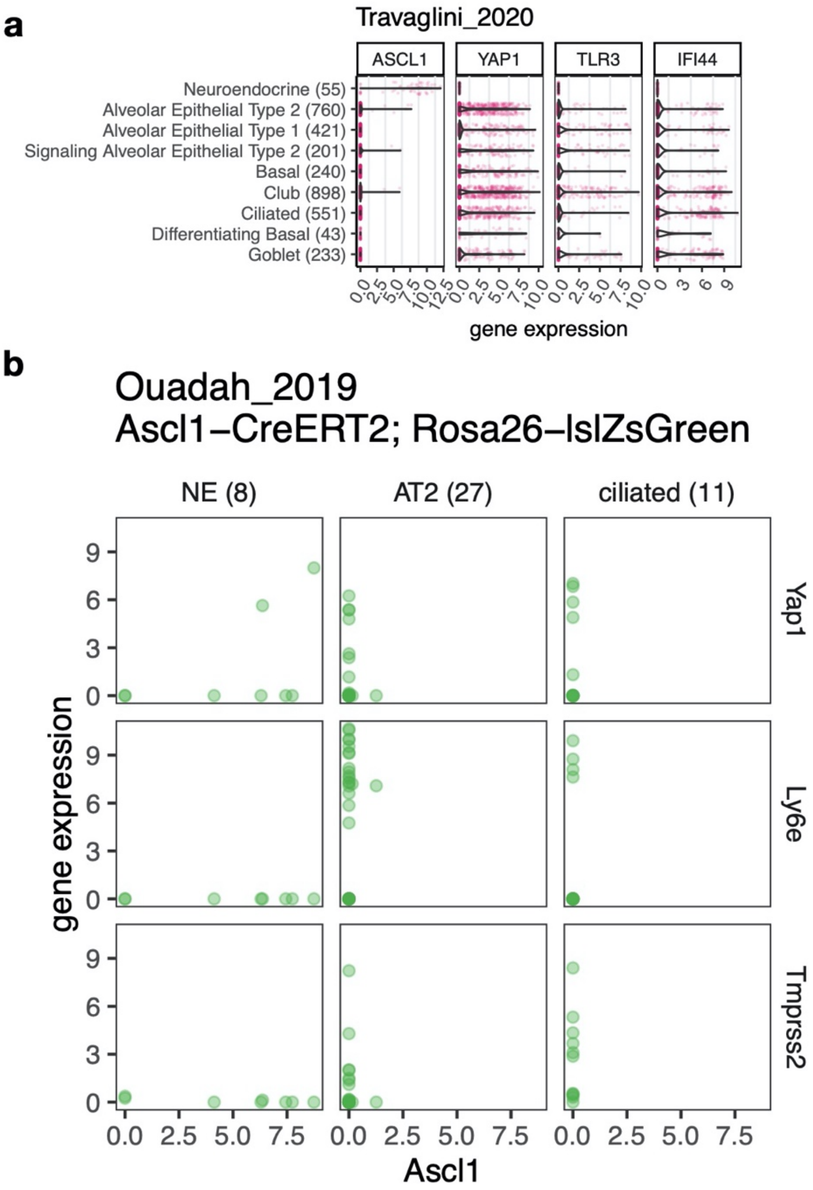

Supplementary Figure 4 Repression of viral defense/hijacked genes in PNECs compared to other lung epithelial cell types

a, Violin plots showing expression of selected lineage factor (*ASCL1* and *YAP1*) and ISGs (*TLR3* and *IFI44*) in healthy human lung epithelial cells based on scRNA-seq experiments from "Travaglini_2020". Note that from research of Zika virus, *TLR3* has been shown to mediate the deleterious effect of Zika virus through disruption of neurogenesis (5). **b**, Scatter plot showing relationship among selected lineage factor genes (*Ascl1* and *Yap1*) and ISGs (*Ly6e* and *Tmprss2*) in scRNA-seq data ("Ouadah_2019") of lung epithelial cells isolated from a mouse model genetically engineered to enable lineage tracing of PNECs. AT2 and ciliated cells transdifferentiated from PNECs have lost expression of *Ascl1* but upregulated *Yap1*, *Ly6e* and *Tmprss2*. Note that from research of coronavirus, *LY6E* is implicated in viral defense (6) whereas *TMPRSS2* mediates viral entry *(7)*. For Travaglini_2020 data in S3a, library size normalized and log2 transformed count data was used whereas for Ouadah_2019 data in S3b, log2 transformed Transcripts Per Million (TPM) expression data was used.

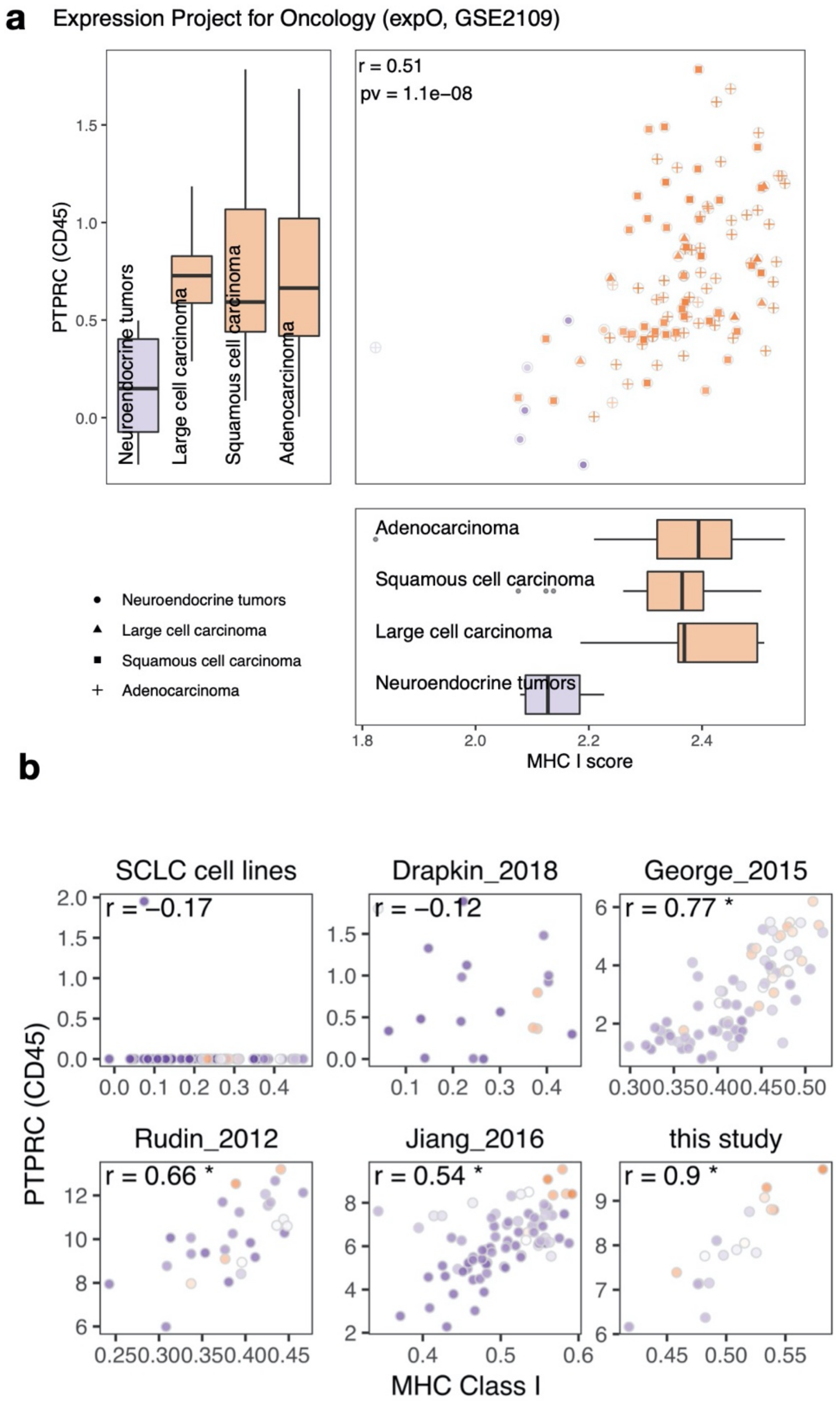

Supplementary Figure 5 Expression of MHC I gene is positively associated with pan-leukocyte marker CD45 (encoded by *PTPRC*) in lung tumors and SCLC tumors

a, Expression of MHC I genes and *PTPRC* in lung tumors from Expression Project for Oncology (expO). Center line in boxplot represents median, the lower and upper hinges correspond to the first and third quartiles, the whiskers extends from the hinge to the value no further than 1.5 * IQR away. **b**,Positive correlation between MHC I expression scores and *PTPRC* in SCLC tumors but not cell lines or PDXs ("Drapkin_2018"). *, p-value < 0.05. MHC I scores were calculated by ssGSEA method.

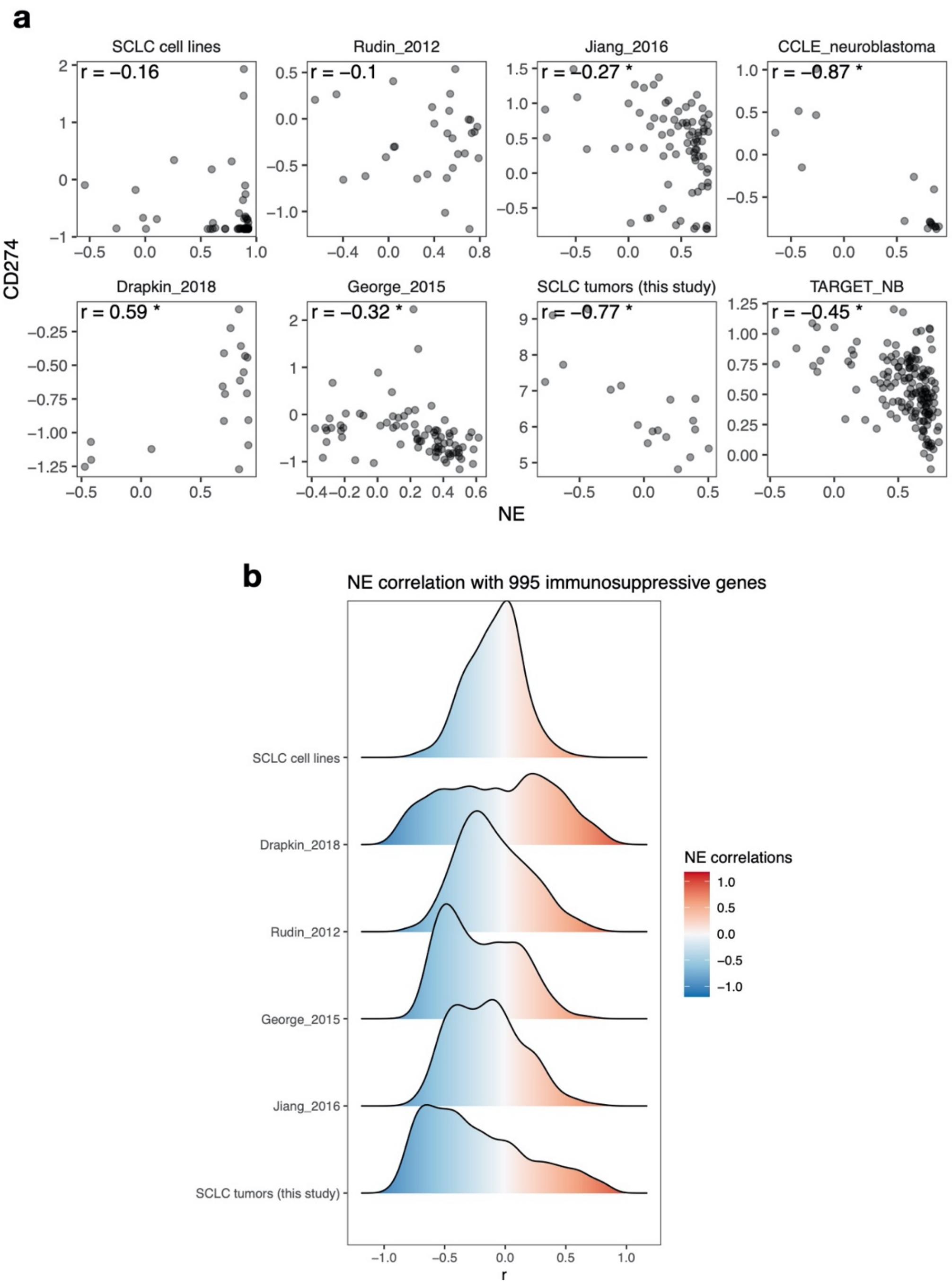

Supplementary Figure 6 Upregulation of immunosuppressive genes in low NE-score SCLC tumors

a, Correlation between *CD274* (PD-L1) expression and NE score. Negative association was not observed in SCLC cell line or PDX ("Drapkin_2018") datasets but was observed in 3 out of 4 SCLC primary tumor datasets as well as NBL cell line (CCLE_neuroblastoma) and tumor (TARGET_NB) datasets. *, Pearson correlation with p-value < 0.05. **b**, Ridgeline plot showing distribution of Pearson correlation coefficients from correlating NE score to expression of 995 immunosuppressive genes (8) in SCLC cell line, PDX and patient tumor datasets. With meta-analysis in the four SCLC tumor datasets, 562 out of the 995 genes were found to have significant correlation with NE score and about 80% of those are negative correlations.

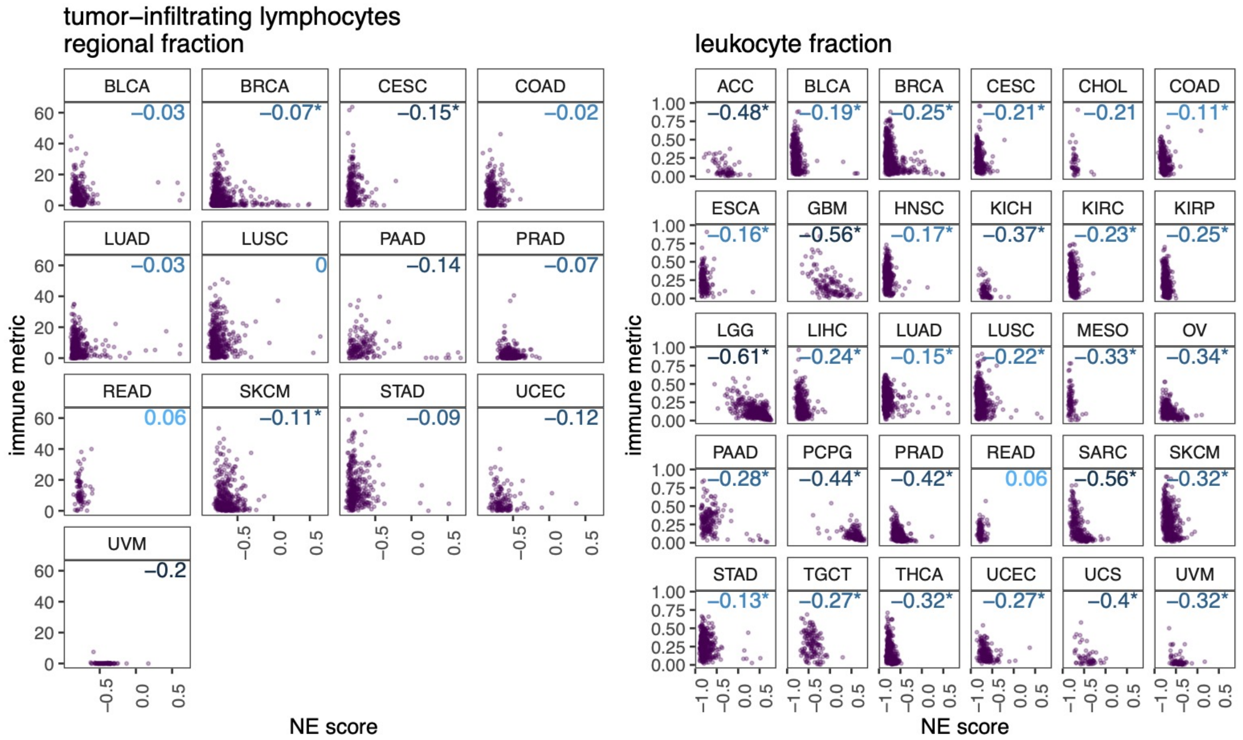

Supplementary Figure 7 Cancer type-specific association between NE score and immune cell fractions

Cancer type-specific scatter plots of NE scores with tumor-infiltrating lymphocytes regional fraction (**a**) or leukocyte fraction (**b**) estimated by Thorsson, Gibbs, et al. (9) in TCGA samples.

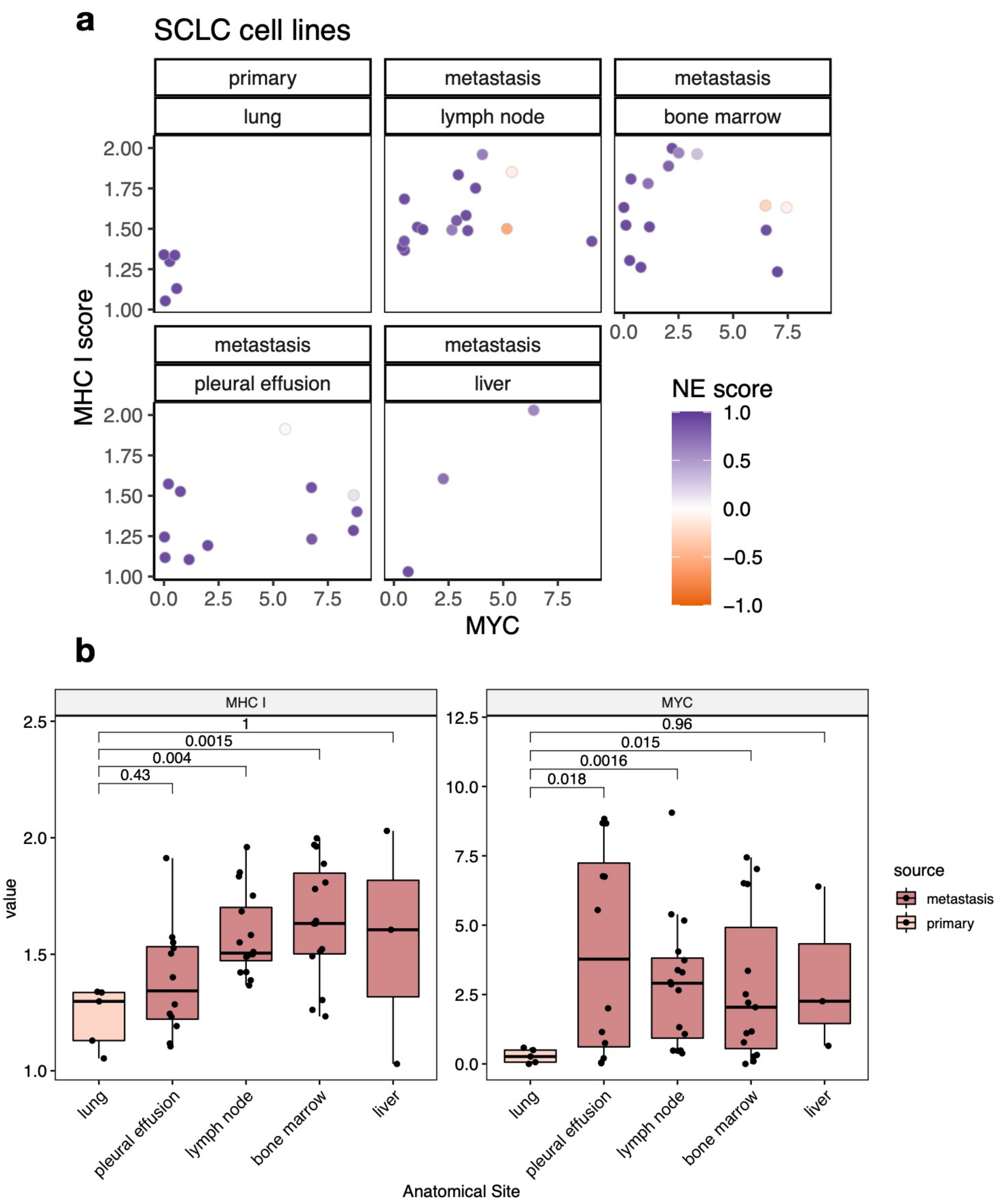

Supplementary Figure 8 Expression of *MYC* and MHC I genes (summarized as MHC I scores) in SCLC cell lines derived from primary lung tumors and metastatic tumors. **a**, Scatterplots of MHC I score and *MYC* expression in lung cancer cell lines by samples types and anatomical sites of origin. **b**, MYC and MHC I gene expression score compared by anatomical site. For each gene, p-values for pairwise comparisons were calculated based on two-sided t-test followed by adjustment for multiple comparisons using the Bonferroni method. Center line in boxplot represents median, the lower and upper hinges correspond to the first and third quartiles, the whiskers extends from the hinge to the value no further than 1.5 * IQR away.

Gene Symbol	Forward Sequence (5'-3')	Reverse Sequence (5'-3')
PSMB8	CACGCTCGCCTTCAAGTTC	AGGCACTAATGTAGGACCCAG
PPIA	CCCACCGTGTTCTTCGACATT	GGACCCGTATGCTTTAGGATGA
B2M	GAGGCTATCCAGCGTACTCCA	CGGCAGGCATACTCATCTTTT
ASCL1	CCTGGTGCGAATGGACTT	CAACGCCACTGACAAGAAAG

Supplementary Table 1. Primer sequences used for this study

Supplementary References

1. Canadas I, Thummalapalli R, Kim JW, Kitajima S, Jenkins RW, Christensen CL, Campisi M, Kuang Y, Zhang Y, Gjini E, Zhang G, Tian T, Sen DR, Miao D, Imamura Y, Thai T, Piel B, Terai H, Aref AR, Hagan T, Koyama S, Watanabe M, Baba H, Adeni AE, Lydon CA, Tamayo P, Wei Z, Herlyn M, Barbie TU, Uppaluri R, Sholl LM, Sicinska E, Sands J, Rodig S, Wong KK, Paweletz CP, Watanabe H, Barbie DA. Tumor innate immunity primed by specific interferon-stimulated endogenous retroviruses. Nat Med. 2018;24(8):1143-50. Epub 2018/07/25. doi: 10.1038/s41591-018-0116-5. PubMed PMID: 30038220; PMCID: PMC6082722.

2. Aran D, Lasry A, Zinger A, Biton M, Pikarsky E, Hellman A, Butte AJ, Ben-Neriah Y. Widespread parainflammation in human cancer. Genome Biol. 2016;17(1):145. Epub 2016/07/09. doi: 10.1186/s13059-016-0995-z. PubMed PMID: 27386949; PMCID: PMC4937599.

3. Ruscetti M, Leibold J, Bott MJ, Fennell M, Kulick A, Salgado NR, Chen CC, Ho YJ, Sanchez-Rivera FJ, Feucht J, Baslan T, Tian S, Chen HA, Romesser PB, Poirier JT, Rudin CM, de Stanchina E, Manchado E, Sherr CJ, Lowe SW. NK cell-mediated cytotoxicity contributes to tumor control by a cytostatic drug combination. Science. 2018;362(6421):1416-22. Epub 2018/12/24. doi: 10.1126/science.aas9090. PubMed PMID: 30573629; PMCID: PMC6711172.

4. Breuer K, Foroushani AK, Laird MR, Chen C, Sribnaia A, Lo R, Winsor GL, Hancock RE, Brinkman FS, Lynn DJ. InnateDB: systems biology of innate immunity and beyond--recent updates and continuing curation. Nucleic Acids Res. 2013;41(Database issue):D1228-33. Epub 2012/11/28. doi: 10.1093/nar/gks1147. PubMed PMID: 23180781; PMCID: PMC3531080.

5. Dang J, Tiwari SK, Lichinchi G, Qin Y, Patil VS, Eroshkin AM, Rana TM. Zika Virus Depletes Neural Progenitors in Human Cerebral Organoids through Activation of the Innate Immune Receptor TLR3. Cell Stem Cell. 2016;19(2):258-65. Epub 2016/05/11. doi: 10.1016/j.stem.2016.04.014. PubMed PMID: 27162029; PMCID: PMC5116380.

6. Pfaender S, Mar KB, Michailidis E, Kratzel A, Hirt D, V'kovski P, Fan W, Ebert N, Stalder H, Kleine-Weber H, Hoffmann M, Hoffmann HH, Saeed M, Dijkman R, Steinmann E, Wight-Carter M, Hanners NW, Pöhlmann S, Gallagher T, Todt D, Zimmer G, Rice CM, Schoggins JW, Thiel V. LY6E impairs coronavirus fusion and confers immune control of viral disease. bioRxiv. 2020.

7. Shen LW, Mao HJ, Wu YL, Tanaka Y, Zhang W. TMPRSS2: A potential target for treatment of influenza virus and coronavirus infections. Biochimie. 2017;142:1-10. Epub 2017/08/06. doi: 10.1016/j.biochi.2017.07.016. PubMed PMID: 28778717; PMCID: PMC7116903.

8. Liu Y, He M, Wang D, Diao L, Liu J, Tang L, Guo S, He F, Li D. HisgAtlas 1.0: a human immunosuppression gene database. Database (Oxford). 2017;2017. Epub 2017/01/01. doi: 10.1093/database/bax094. PubMed PMID: 31725860.

9. Thorsson V, Gibbs DL, Brown SD, Wolf D, Bortone DS, Ou Yang TH, Porta-Pardo E, Gao GF, Plaisier CL, Eddy JA, Ziv E, Culhane AC, Paull EO, Sivakumar IKA, Gentles AJ, Malhotra R, Farshidfar F, Colaprico A, Parker JS, Mose LE, Vo NS, Liu J, Liu Y, Rader J, Dhankani V, Reynolds SM, Bowlby R, Califano A, Cherniack AD, Anastassiou D, Bedognetti D, Mokrab Y, Newman AM, Rao A, Chen K, Krasnitz A, Hu H, Malta TM, Noushmehr H, Pedamallu CS, Bullman S, Ojesina AI, Lamb A, Zhou W, Shen H, Choueiri TK, Weinstein JN, Guinney J, Saltz J, Holt RA, Rabkin CS, Cancer Genome Atlas Research N, Lazar AJ, Serody JS, Demicco EG, Disis ML, Vincent BG, Shmulevich I. The Immune Landscape of Cancer. Immunity. 2018;48(4):812-30 e14. Epub 2018/04/10. doi: 10.1016/j.immuni.2018.03.023. PubMed PMID: 29628290; PMCID: PMC5982584.
