# Supplementary material for: Cell-autonomous immune gene expression is repressed in pulmonary neuroendocrine cells and small cell lung cancer
Source: Commun Biol. 2021 Mar 9;4:314. doi: 10.1038/s42003-021-01842-7 (PMC7943563; doi:10.1038/s42003-021-01842-7)
Supplement: Supplementary file 3 — Description of Additional Supplementary Files [file 42003_2021_1842_MOESM3_ESM.pdf]

Description of Additional Supplementary Files

File name: Supplementary Data 1-5

File Description:

Supplementary Data 1 - NE signature based on SCLC cell line RNA-seq data

Supplementary Data 2 - Patient characteristics, data availability and quantification of CD4 and CD8 T cells based on IHC

Supplementary Data 3 - GSEA results for SCLC cell lines and George_2015 tumor datasets.

Supplementary Data 4 - Correlation between genes from InnateDB and NE scores in SCLC and NBL cell lines

Supplementary Data 5 - Correlation between 995 immunosuppressive genes and NE scores from SCLC datasets
