# Supplementary material for: Cell-autonomous immune gene expression is repressed in pulmonary neuroendocrine cells and small cell lung cancer
Source: Commun Biol. 2021 Mar 9;4:314. doi: 10.1038/s42003-021-01842-7 (PMC7943563; doi:10.1038/s42003-021-01842-7)
Supplement: Supplementary file 5 — Reporting Summary [file 42003_2021_1842_MOESM5_ESM.pdf]

nature research

Corresponding author(s): Ling Cai
John Minna
Yang Xie

Last updated by author(s): Jan 23, 2021

Reporting Summary

Nature Research wishes to improve the reproducibility of the work that we publish. This form provides structure for consistency and transparency in reporting. For further information on Nature Research policies, see our Editorial Policies and the Editorial Policy Checklist.

Statistics

For all statistical analyses, confirm that the following items are present in the figure legend, table legend, main text, or Methods section.

n/a	Confirmed	
☐	☒	The exact sample size (*n*) for each experimental group/condition, given as a discrete number and unit of measurement
☐	☒	A statement on whether measurements were taken from distinct samples or whether the same sample was measured repeatedly
☐	☒	The statistical test(s) used AND whether they are one- or two-sided<br>*Only common tests should be described solely by name; describe more complex techniques in the Methods section.*
☒	☐	A description of all covariates tested
☐	☒	A description of any assumptions or corrections, such as tests of normality and adjustment for multiple comparisons
☐	☒	A full description of the statistical parameters including central tendency (e.g. means) or other basic estimates (e.g. regression coefficient) AND variation (e.g. standard deviation) or associated estimates of uncertainty (e.g. confidence intervals)
☐	☒	For null hypothesis testing, the test statistic (e.g. *F*, *t*, *r*) with confidence intervals, effect sizes, degrees of freedom and *P* value noted<br>*Give P values as exact values whenever suitable.*
☒	☐	For Bayesian analysis, information on the choice of priors and Markov chain Monte Carlo settings
☒	☐	For hierarchical and complex designs, identification of the appropriate level for tests and full reporting of outcomes
☐	☒	Estimates of effect sizes (e.g. Cohen's *d*, Pearson's *r*), indicating how they were calculated

Our web collection on statistics for biologists contains articles on many of the points above.

Software and code

Policy information about availability of computer code

Data collection	We provide the accession code and sources of public data used in our study in the material and methods section of our manuscript. Microarray data generated for this study has been deposited to GEO with accession id GSE149507, RNA-seq data has been deposited to dbGaP with accession id phs001823.v1.p1. Input data for running the scripts to generate figures used in this paper has been deposited to Dryad Digital Repository (https://datadryad.org/stash/share/BkmPdMrwhae1VxhkkSLIG_532FLCqcYiMFUpY1yKmGA).
Data analysis	All the data analysis were performed with R. The R packages used for different analyses have been described in the material and methods section of our manuscript. We have also deposited our scripts to Dryad Digital Repository (https://datadryad.org/stash/share/BkmPdMrwhae1VxhkkSLIG_532FLCqcYiMFUpY1yKmGA).

For manuscripts utilizing custom algorithms or software that are central to the research but not yet described in published literature, software must be made available to editors and reviewers. We strongly encourage code deposition in a community repository (e.g. GitHub). See the Nature Research guidelines for submitting code & software for further information.

Data

Policy information about availability of data

All manuscripts must include a data availability statement. This statement should provide the following information, where applicable:

- Accession codes, unique identifiers, or web links for publicly available datasets
- A list of figures that have associated raw data
- A description of any restrictions on data availability

The RNA-seq gene expression data from UTSW SCLC has been added to dbGaP (accession phs001823.v1.p1) (101). SCLC tumor microarray data used in this study has been deposited to GEO with accession id GSE149507.

Field-specific reporting

Please select the one below that is the best fit for your research. If you are not sure, read the appropriate sections before making your selection.

- [x] Life sciences
- [ ] Behavioural & social sciences
- [ ] Ecological, evolutionary & environmental sciences

For a reference copy of the document with all sections, see nature.com/documents/nr-reporting-summary-flat.pdf

Life sciences study design

All studies must disclose on these points even when the disclosure is negative.

Sample size	We did not perform calculation of sample size
Data exclusions	We did not exclude data
Replication	We collected multiple published datasets and generated our own dataset to perform our analyses to ensure the highest reproducibility.
Randomization	For the xenograft tumor growth experiments, mice were randomized before allocated to cages for treatment.
Blinding	Investigator was blinded during the xenograft treatment experiments.

Reporting for specific materials, systems and methods

We require information from authors about some types of materials, experimental systems and methods used in many studies. Here, indicate whether each material, system or method listed is relevant to your study. If you are not sure if a list item applies to your research, read the appropriate section before selecting a response.

Materials & experimental systems

n/a	Involved in the study	
☐	☒	Antibodies
☐	☒	Eukaryotic cell lines
☒	☐	Palaeontology and archaeology
☐	☒	Animals and other organisms
☒	☐	Human research participants
☒	☐	Clinical data
☒	☐	Dual use research of concern

Methods

n/a	Involved in the study	
☒	☐	ChIP-seq
☒	☐	Flow cytometry
☒	☐	MRI-based neuroimaging

Antibodies

Antibodies used	ASCL1 (dilution 1:25; Clone 24B72D11.1, BD Biosciences, Catalog # 556604), NEUROD1 (dilution 1:100; Clone EPR20766, Abcam, ab213725), POU2F3 (dilution 1:200; polyclonal, Novus Biologicals, NBP1-83966), CD4 (dilution 1:80; Leica Biosystems, CD4-368-L-CE-H) and CD8 (dilution 1:25; Thermo Scientific, MS-457s)
Validation	FFPE cell lines pellets with known expression of ASCL1, NEUROD1 and POU2F3 were used to establish and optimize IHC conditions and assess sensitivity and specificity for each antibody.

Eukaryotic cell lines

Policy information about cell lines

Cell line source(s)	All SCLC cell lines used in these studies were originally established in the John D. Minna and Adi F. Gazdar laboratories. The cultured Small Cell Lung Cancer (SCLC) cell lines were obtained from both the National Cancer Institute (NCI) and Hamon Cancer Center (HCC) libraries.
Authentication	All cell lines were regularly fingerprinted using a PowerPlex 1.2 kit (Promega, Madison, WI) to confirm the cell line identity
Mycoplasma contamination	All cell lines were regularly tested for mycoplasma contamination (Bulldog Bio, Portsmouth, NH).
Commonly misidentified lines (See ICLAC register)	*Name any commonly misidentified cell lines used in the study and provide a rationale for their use.*

Animals and other organisms

Policy information about studies involving animals; ARRIVE guidelines recommended for reporting animal research

Laboratory animals	6-8-week-old NSG mice (Jackson Laboratory #005557) were used for the study
Wild animals	The study does not involve wild animals.
Field-collected samples	The study does not involve filed-collected samples.
Ethics oversight	All mouse procedures were performed with the approval of the University of Texas Southwestern Medical Center IACUC.

Note that full information on the approval of the study protocol must also be provided in the manuscript.
